# Normocalcemic Presentation of a Giant Polycystic Parathyroid Carcinoma

**DOI:** 10.1210/jcemcr/luaf077

**Published:** 2025-04-18

**Authors:** Keito Ichinohe, Takashi Nishi, Shintaro Goto, Kaori Kameyama, Yukihiro Fujita, Takeshi Nigawara

**Affiliations:** Department of Endocrinology and Metabolism, Tsugaru General Hospital, Goshogawara, Aomori 037-0074, Japan; Department of Endocrinology and Metabolism, Hirosaki University Graduate School of Medicine, Hirosaki, Aomori 036-8562, Japan; Department of Mammary and Thyroid Surgery, Yushinkai Aomori Shintoshi Hospital, Aomori, Aomori 038-0003, Japan; Department of Pathology and Bioscience, Hirosaki University Graduate School of Medicine, Hirosaki, Aomori 036-8562, Japan; Department of Pathology, Showa University Northern Yokohama Hospital, Yokohama, Kanagawa 224-8503, Japan; Department of Endocrinology and Metabolism, Hirosaki University Graduate School of Medicine, Hirosaki, Aomori 036-8562, Japan; Department of Endocrinology and Metabolism, Tsugaru General Hospital, Goshogawara, Aomori 037-0074, Japan

**Keywords:** parathyroid carcinoma, normocalcemia, polycystic, fine needle aspiration cytology, tumor deposit

## Abstract

A 70-year-old woman presented with a large anterior cervical mass. Computed tomography and ultrasonography revealed a 70-mm polycystic structure adjacent to the right lobe of the thyroid gland, extending to its caudal aspect. Serum calcium and TSH levels were normal, whereas the serum intact PTH level was slightly elevated. Fine-needle aspiration showed cytology findings consistent with nodular goiter. After 20 months, the tumor was smaller on ultrasound but was completely solid. Serum PTH levels were markedly elevated, and Tc-99m sestamibi scintigraphy revealed striking hyperactivity of the mass. The patient underwent right hemithyroidectomy with en bloc tumor resection. Pathological analysis revealed capsular, thyroid, and venous invasion. Immunohistochemical staining was positive for PTH and galectin-3, and a high mitotic index was observed. Based on these findings, parathyroid carcinoma was diagnosed. Parafibromin staining was positive, resulting in the exclusion of etiology associated with *CDC73* pathogenic variants. After 10 months, the patient was clinically free of recurrent disease. This case illustrates an atypical presentation of parathyroid carcinoma with early normocalcemia, followed by overt hyperparathyroidism, possibly because of tumor transformation.

## Introduction

Parathyroid carcinoma (PC) is the rarest type of endocrine malignancy [1]. It typically results in a marked increase in serum calcium and PTH levels. Here, we describe a case of PC that initially presented with normocalcemia and only a slight increase in serum PTH levels, despite a massive tumor with an atypical appearance. Fine-needle aspiration cytology (FNAC) performed before the onset of hypercalcemia indicated a nodular goiter (NG), which delayed the diagnosis.

## Case Presentation

A 70-year-old woman presented to our hospital with a large anterior cervical mass that had been gradually growing for 6 months. The patient did not report any local pain, dysphagia, hoarseness, or weight loss. Her medical history was noncontributory, apart from depression, which was under medication, and uterine prolapse, which was managed with a device. The patient did not have a personal or family history of urolithiasis, pathological fractures, endocrine tumors, or jaw tumors.

## Diagnostic Assessment

**Figure 1.**
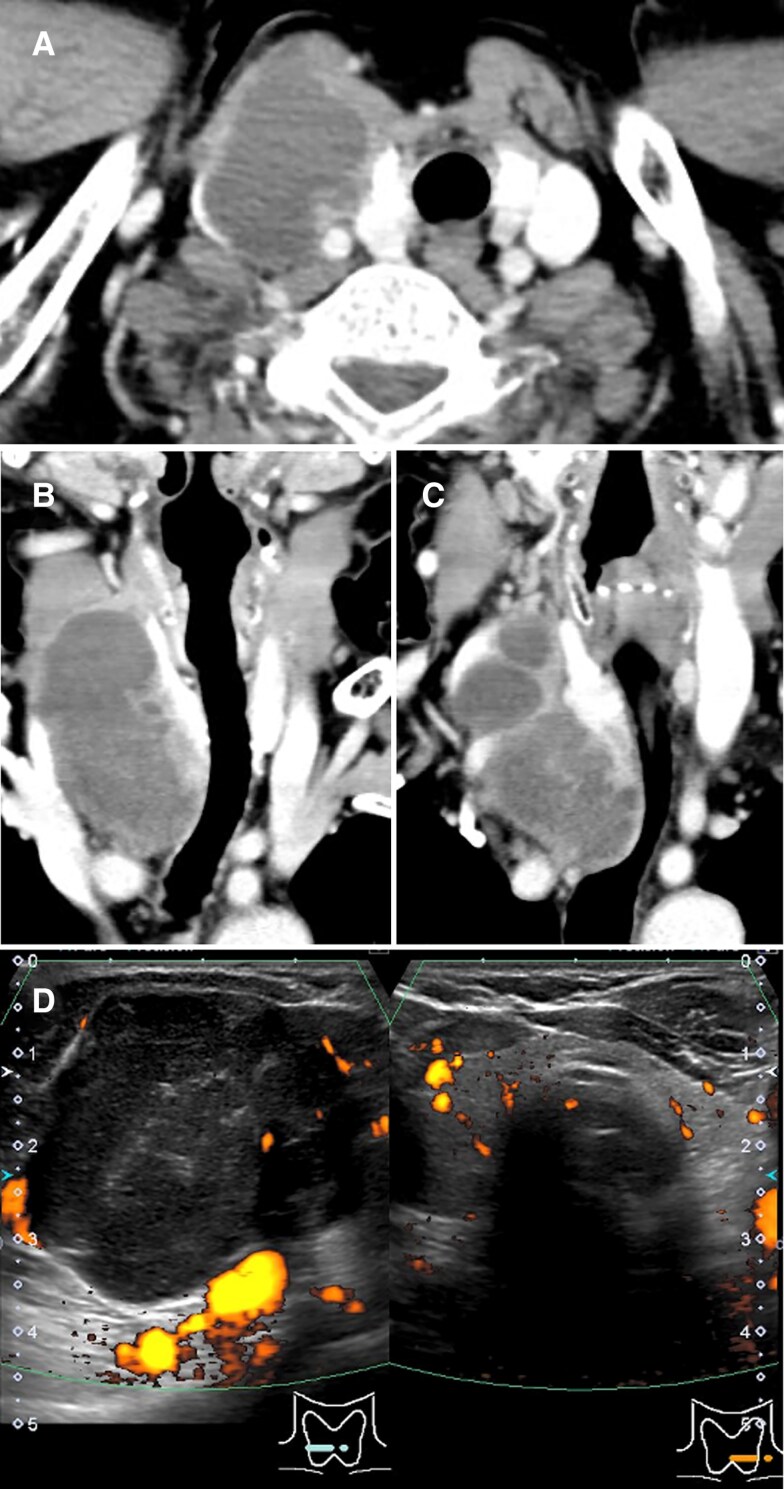
Cervical computed tomography (A, B, C) and color Doppler ultrasonography (D) at presentation. The main locus of the polycystic tumor was the lateral aspect of the thyroid gland.

Physical examination revealed a multinodular, rubber-hard, mobile, and nontender mass. Laboratory tests revealed a slight increase in alkaline phosphatase, thyroglobulin, and intact PTH levels (Table 1). The serum calcium level was normal. A minor decrease in the free thyroxine level along with positivity for antithyroid peroxidase antibody suggested occult Hashimoto thyroiditis. Computed tomography revealed a multiloculated cystic mass measuring 70 × 46 × 35 mm, located adjacent to the right thyroid lobe and extending to its caudal aspect. The mass displaced the common carotid artery posteriorly and the internal jugular vein laterally (Fig. 1A-1C). No lymphadenopathy or distant metastasis was observed. The mass appeared hypoechoic and heterogeneous and lacked a blood flow signal (Fig. 1D). Ultrasonography-guided fine-needle aspiration collected 15 mL of reddish serous fluid. FNAC revealed epithelial cells without atypia that formed small follicular clusters, consistent with NG.

**Table 1.** Time course of serum parameters

Parameters	At presentation	20 months later	Normal reference range
Albumin	4.0 g/dL	4.0 g/dL	4.0-5.1 g/dL
	(40 g/L)	(40 g/L)	(40-51 g/L)
Calcium	9.9 mg/dL	**14.6 mg/dL**	8.8-10.1 mg/dL
	(2.47 mmol/L)	**(3.64 mmol/L)**	(2.20-2.52 mmol/L)
Phosphate	3.0 mg/dL	**2.3 mg/dL**	2.6-5.5 mg/dL
	(0.97 mmol/L)	**(0.74 mmol/L)**	(0.84-1.78 mmol/L)
Alkaline phosphatase	**127 U/L**	**238 U/L**	38-113 U/L
	**(2120 nkat/L)**	**(3970 nkat/L)**	(630-1880 nkat/L)
Urea nitrogen	19.4 mg/dL	10.9 mg/dL	8.0-20.0 mg/dL
	(6.93 mmol/L)	(3.89 mmol/L)	(2.86-7.14 mmol/L)
Creatinine	0.73 mg/dL	0.87 mg/dL	0.46-0.79 mg/dL
	(64.55 μmol/L)	(76.93 μmol/L)	(40.67-69.85 μmol/L)
Free triiodothyronine	2.66 pg/mL	2.70 pg/mL	2.13-4.07 pg/mL
	(4.09 pmol/L)	(4.15 pmol/L)	(3.27-6.25 pmol/L)
Free thyroxine	**0.85 ng/dL**	**0.92 ng/dL**	0.95-1.70 ng/dL
	**(10.94 pmol/L)**	**(11.84 pmol/L)**	(12.23-21.88 pmol/L)
TSH	3.050 μIU/mL	2.478 μIU/mL	0.610-4.230 μIU/mL
	(3.050 mIU/L)	(2.478 mIU/L)	(0.610-4.230 mIU/L)
Thyroglobulin	**47.0 ng/mL**	27.5 ng/mL	<35.1 ng/mL
	**(6.05 pmol/L)**	(3.54 pmol/L)	(<4.52 pmol/L)
TPOAb	**3.9 IU/mL**	**4.0 IU/mL**	<3.0 IU/mL
	**(3.9 kIU/L)**	**(4.0 kIU/L)**	(<3.0** **kIU/L)
TgAb	<10.0 IU/mL	<10.0 IU/mL	<19.0 IU/mL
	(<10.0 kIU/L)	(<10.0 kIU/L)	(<19.0 kIU/L)
Intact PTH	**69 pg/mL**	**394 pg/mL**	<65 pg/mL
	**(7.32 pmol/L)**	**(41.78 pmol/L)**	(<6.89 pmol/L)

Abnormal values are shown in bold font.

Abbreviations: TgAb, antithyroglobulin antibody; TPOAb, antithyroid peroxidase antibody.

**Figure 2.**
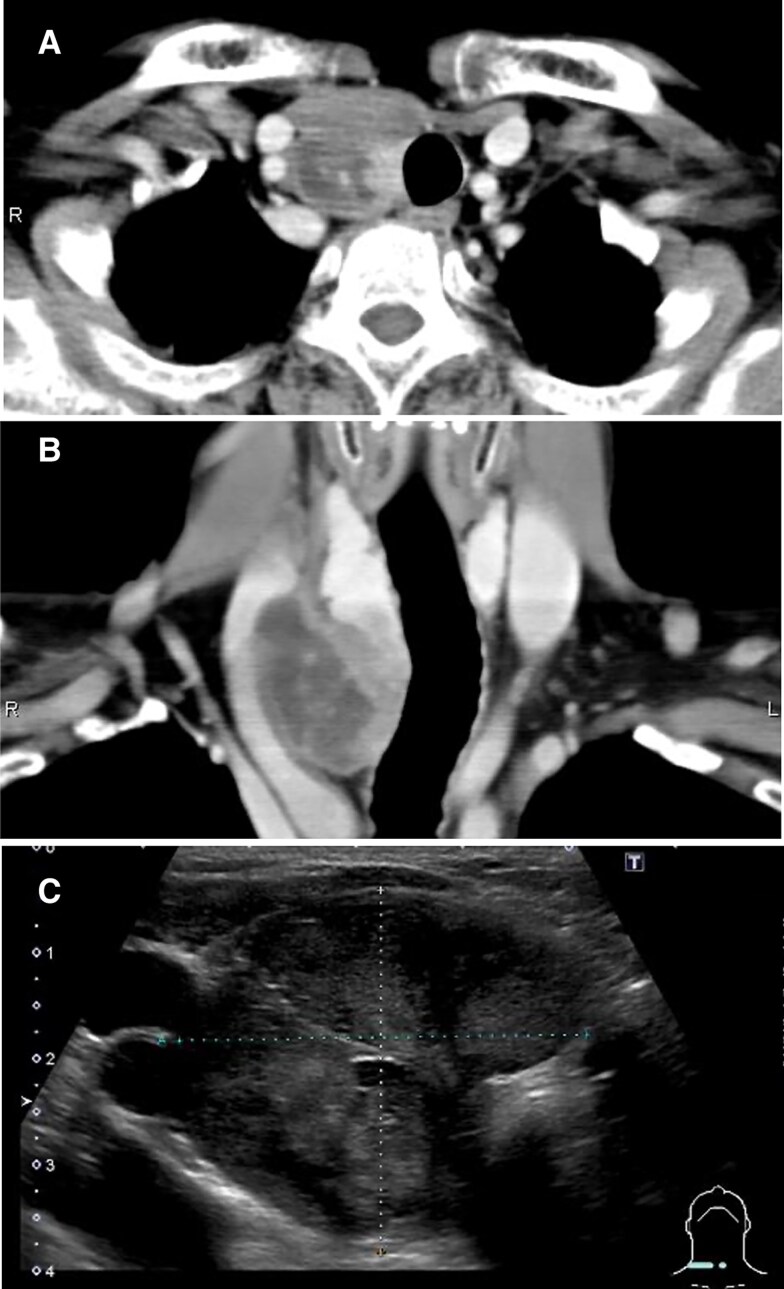
Cervical computed tomography (A, B) and ultrasonography (C) 7 months after presentation. Shrinkage of the cystic components is noted.

Seven months after presentation, computed tomography and ultrasonography revealed shrinkage of the cystic component, which had converged in the caudal direction (Fig. 2A-2C). Despite having an appointment 3 months later, the patient was lost to follow-up.

**Figure 3.**
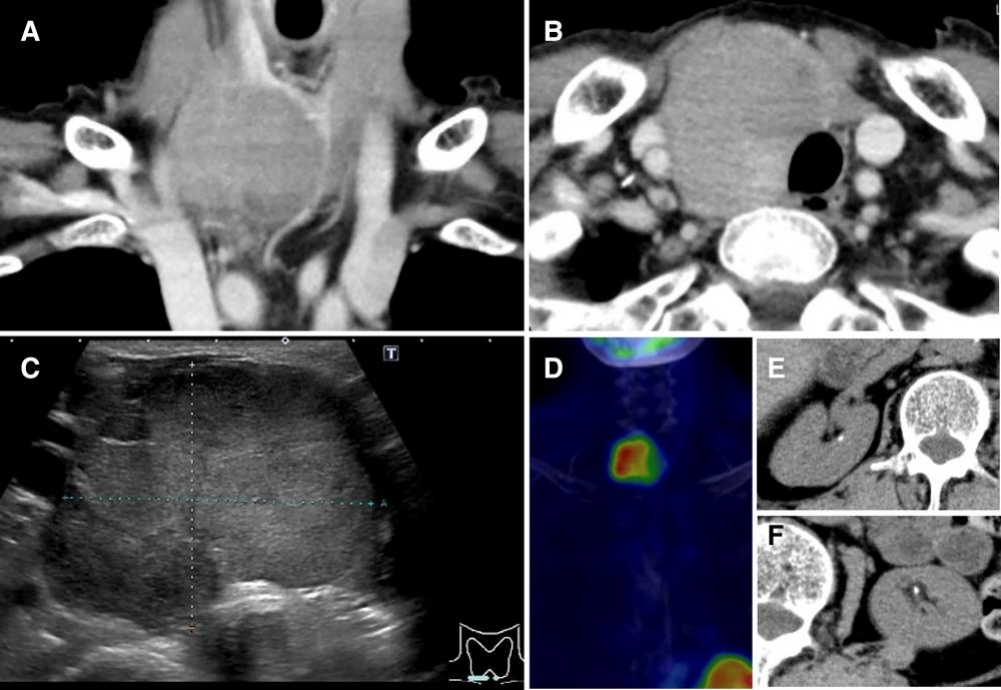
Twenty months after presentation, the tumor became almost totally solid on computed tomography (CT) (A, B) and ultrasonography (C) and was positive for Tc-99 m sestamibi scintigraphy (D). Small renal calculi were noted on CT (E, F).

Twenty months after the presentation, the patient was referred to our hospital from her psychiatry clinic for a workup of electrolyte disturbance. Marked hypercalcemia concurrent with hypophosphatemia was attributed to a strikingly elevated serum intact PTH level (Table 1). The patient did not complain of thirst, polydipsia, or weight loss. The tumor grew in size from 40 to 52 mm in diameter and was composed entirely of solid components (Fig. 3A-3C). The positive uptake of Tc-99m sestamibi was consistent with a parathyroid tumor (Fig. 3D). No distant metastases were observed. Small renal calculi were noted (Fig. 3E and 3F). The compression fractures of the fourth and fifth lumbar vertebrae and a T-score of −4.0 at the femoral neck on dual X-ray absorptiometry indicated overt osteoporosis.

## Treatment

The patient received a single IV dose of 4 mg zoledronate, which successfully reduced the serum calcium level to 11.6 mg/dL (2.89 mmol/L) (normal reference, 8.8-10.1 mg/dL; 2.20-2.52 mmol/L), thereby lowering the risk of hypercalcemic crisis. The patient then underwent en bloc resection of the tumor along with a right hemithyroidectomy.

## Outcome and Follow-up

**Figure 4.**
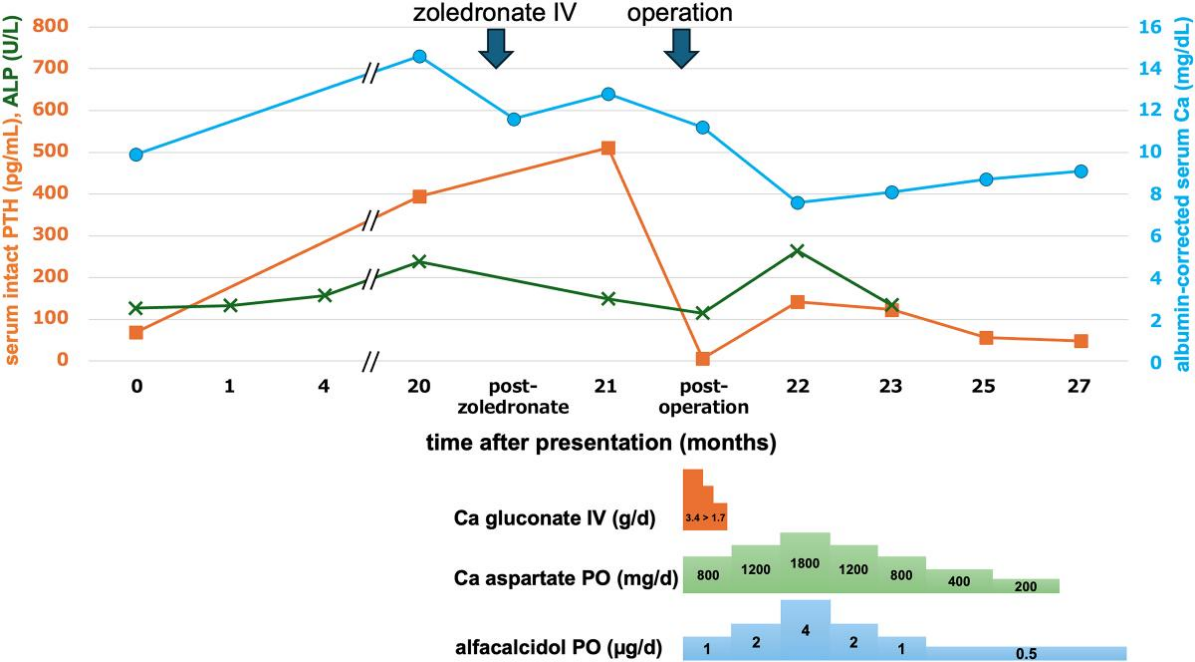
Time course of serum parameters in reference to replacement dosage. Intact PTH levels decreased immediately after surgery, whereas calcium supplementation maintained the albumin-corrected calcium level. Abbreviation: ALP, alkaline phosphatase.

**Figure 5.**
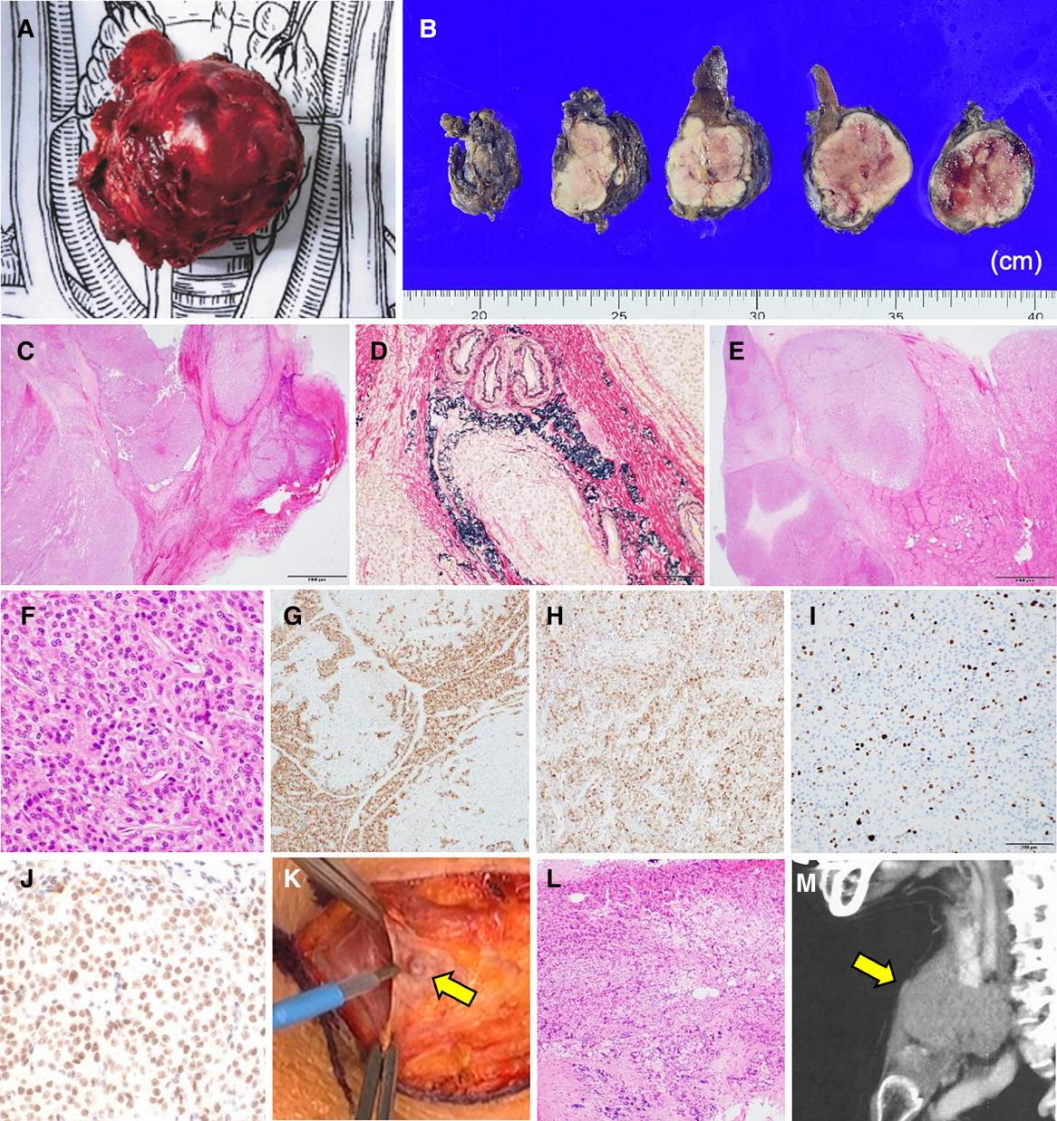
(A, B) Macroscopic images of the surgical specimen. (C) Capsular invasion (magnification: 40×). (D) Venous invasion (200×). (E) Thyroid invasion of the tumor (40×). (F) Stippled nucleoli (400×). (G) Partially positive PTH staining (100×). (H) Diffusely positive galectin-3 staining (100×). (I) Ki-67 index was 17% (200×). (J) Positive parafibromin staining (400×). (K) A pea-sized nodule on the surface of the platysma. (L) Tumor deposit without lymphatic component (200×). (M) The location of the tumor deposit on computed tomography. (C, E, F, L) Hematoxylin-eosin staining. (D) Elastica-van Gieson staining.

Following surgery, serum calcium and intact PTH levels returned to normal after the “hungry-bone” phase, with maintenance treatment consisting of alfacalcidol and calcium aspartate (Fig. 4). Although the tumor was highly adhesive to the surrounding soft tissues, the surgical margin was histologically negative (macroscopic view: Fig. 5A and 5B). The tumor exhibited capsular, venous, and thyroid invasion (Fig. 5C-5E). The tumor cells, characterized by slightly eosinophilic cytoplasm, oval nuclei, and small nucleoli, were arranged in a trabecular pattern (Fig. 5F). The cells were partially positive for PTH and diffusely positive for galectin-3 immunostaining (Fig. 5G and 5H). The Ki-67 index was 17% (Fig. 5I). Therefore, a diagnosis of PC was confirmed [2]. Parafibromin staining was positive, resulting in the exclusion of etiology associated with *CDC73* pathogenic variants (Fig. 5J).

A pea-sized nodule on the surface of the platysma noted during surgery (Fig. 5K) was a tumor deposit without a lymphatic component (Fig. 5L). A computed tomography review revealed a small subcutaneous nodule located anterior to the tumor (Fig. 5M). No local recurrence or distant metastases were observed 10 months after surgery.

## Discussion

PC is a rare malignancy, accounting for <1% of primary hyperparathyroidism cases and approximately 0.005% of all cancers [1]. It is typically associated with severe symptomatic hypercalcemia; however, in the current case, the patient was normocalcemic at the time of the first presentation. Thirty-three previously reported cases of nonfunctional PC remained permanently nonfunctional [3], whereas the current case eventually became functional. Considering the polycystic appearance of the tumor, we speculate that this case may have presented during a normocalcemic window following a degenerative event of the neoplasm, although the ictus was not apparent in the clinical course. Although osteoporosis and urolithiasis in this patient may reflect the disease duration of PC, it is difficult to accurately assess their contribution, as both complications are common in elderly women in the general population [4].

The location and morphology of the tumor were also puzzling in terms of diagnosis. Initial imaging studies provided the impression that the primary tumor site was in the lateral aspect of the thyroid gland; however, the studies conducted 7 months later suggested that it likely originated in the caudal aspect of the thyroid gland, consistent with the right lower parathyroid gland. Only a few PCs exhibit a cystic appearance [5]. To the best of our knowledge, no imaging studies have reported cases of polycystic PC mimicking the morphological phenotype of NG.

Based on the morphological changes in the tumor, it appeared that PC may have replaced the preexisting NG; however, alternative perspectives exist. Although a typical NG shows a deformity affecting the entire thyroid gland [6], the resected thyroid tissue that was spared from tumor invasion did not demonstrate any nodular changes (Fig. 5E). The initial imaging studies confirmed that most of the right lobe was intact (Fig. 1A-1D). No NG fragments were identified within the resected tumor (Fig. 5B-5E). The complete replacement of NG by PC confined to the right lobe, along with only partial invasion of the normal right lobe, appears to represent an unusual pattern of tumor growth. Considering the diagnostic limitations of FNAC discussed next, it is more likely that the tumor was a PC at presentation rather than an NG.

Aside from a few exploratory studies [7], many investigations agree on FNAC's unreliability to diagnose parathyroid adenoma without establishing specific cytological features, much less PC [1, 8]. Some PCs exhibit cytological features similar to those of follicular thyroid neoplasms [9, 10], which may be relevant to this case. Alternatively, PTH measurement in the aspirate of benign parathyroid lesions has recently gained popularity [11]. This method has also been applied to nonsecreting parathyroid cysts [12]. Immunohistochemistry of the aspirated material has also been attempted [13].

In contrast to benign lesions, aspiration of PC is not recommended because it risks tumor dissemination [1, 8]. In our case, the subcutaneous tumor deposit may have been seeded during the FNAC procedure, which was performed before the suspicion of PC. Tc-99m sestamibi scintigraphy may help identify the tumor of parathyroid origin but cannot assess malignant potential [14]. F-18 fluorodeoxyglucose positron emission tomography/computed tomography is used to investigate metastasis, rather than to differentiate the malignancy of the primary parathyroid tumor. However, little is known about the diagnostic accuracy of PC [8]. The establishment of noninvasive preoperative diagnostic methods for PC, such as the detection of long noncoding RNA BC200 in the circulating blood, is expected in the future [15].

## Learning Points

- An unusual subtype of PC exists in which hypercalcemia may not be a hallmark of the disease.
- A seemingly nonfunctioning PC can transform into an overtly functioning one. The present tumor may have been insufficiently functional to cause overt hypercalcemia at presentation.
- Some PCs have a polycystic appearance that resembles that of NG.
- FNAC is not recommended for PC. In the absence of overt hyperparathyroidism in a suspicious cervical tumor, careful diagnostic en bloc resection following a qualitative assessment using Tc-99m sestamibi scintigraphy and/or an exploratory evaluation of malignant potential by F-18 fluorodeoxyglucose positron emission tomography/computed tomography may be an appropriate clinical choice, before the advent of a useful circulating biomarker of PC.

## Data Availability

Some or all datasets generated during and/or analyzed during the current study are not publicly available but are available from the corresponding author on reasonable request.

## Abbreviations

FNAC: fine-needle aspiration cytology
NG: nodular goiter
PC: parathyroid carcinoma
